# Individual Brain Tumor Invasion Mapping Based on Diffusion Kurtosis Imaging

**DOI:** 10.17691/stm2025.17.1.08

**Published:** 2025-02-28

**Authors:** E.L. Pogosbekyan, N.E. Zakharova, A.I. Batalov, A.M. Shevchenko, L.M. Fadeeva, A.E. Bykanov, A.N. Tyurina, I.V. Chekhonin, S.A. Galstyan, D.I. Pitskhelauri, I.N. Pronin, D.Yu. Usachev

**Affiliations:** Medical Physicist, Department of Neuroradiology; N.N. Burdenko National Medical Research Center of Neurosurgery of the Ministry of Health of the Russian Federation, 16, 4^th^ Tverskaya-Yamskaya St., Moscow, 125047, Russia; MD, DSc, Professor of the Russian Academy of Sciences, Chief Researcher, Department of Neuroradiology; N.N. Burdenko National Medical Research Center of Neurosurgery of the Ministry of Health of the Russian Federation, 16, 4^th^ Tverskaya-Yamskaya St., Moscow, 125047, Russia; MD, PhD, Researcher, Department of Neuroradiology; N.N. Burdenko National Medical Research Center of Neurosurgery of the Ministry of Health of the Russian Federation, 16, 4^th^ Tverskaya-Yamskaya St., Moscow, 125047, Russia; Radiologist, Department of Neuroradiology; N.N. Burdenko National Medical Research Center of Neurosurgery of the Ministry of Health of the Russian Federation, 16, 4^th^ Tverskaya-Yamskaya St., Moscow, 125047, Russia; Leading Engineer, Department of Neuroradiology; N.N. Burdenko National Medical Research Center of Neurosurgery of the Ministry of Health of the Russian Federation, 16, 4^th^ Tverskaya-Yamskaya St., Moscow, 125047, Russia; MD, PhD, Researcher, Neurosurgery Department No.7; N.N. Burdenko National Medical Research Center of Neurosurgery of the Ministry of Health of the Russian Federation, 16, 4^th^ Tverskaya-Yamskaya St., Moscow, 125047, Russia; MD, PhD, Researcher, Department of Neuroradiology; N.N. Burdenko National Medical Research Center of Neurosurgery of the Ministry of Health of the Russian Federation, 16, 4^th^ Tverskaya-Yamskaya St., Moscow, 125047, Russia; MD, PhD, Radiologist, Department of Neuroradiology; N.N. Burdenko National Medical Research Center of Neurosurgery of the Ministry of Health of the Russian Federation, 16, 4^th^ Tverskaya-Yamskaya St., Moscow, 125047, Russia; Pathologist, Department of Pathology; N.N. Burdenko National Medical Research Center of Neurosurgery of the Ministry of Health of the Russian Federation, 16, 4^th^ Tverskaya-Yamskaya St., Moscow, 125047, Russia; MD, DSc, Professor, Head of Neurosurgery Department No.7; N.N. Burdenko National Medical Research Center of Neurosurgery of the Ministry of Health of the Russian Federation, 16, 4^th^ Tverskaya-Yamskaya St., Moscow, 125047, Russia; MD, DSc, Professor, Academician of the Russian Academy of Sciences, Head of the Department of Neuroradiology; N.N. Burdenko National Medical Research Center of Neurosurgery of the Ministry of Health of the Russian Federation, 16, 4^th^ Tverskaya-Yamskaya St., Moscow, 125047, Russia; MD, DSc, Professor, Academician of the Russian Academy of Sciences, Director; N.N. Burdenko National Medical Research Center of Neurosurgery of the Ministry of Health of the Russian Federation, 16, 4^th^ Tverskaya-Yamskaya St., Moscow, 125047, Russia

**Keywords:** high-grade malignant gliomas, diffusion kurtosis MRI, metastases, tumor invasion maps

## Abstract

**The aim of the investigation** is to develop and implement an algorithm for image analysis in brain tumors (glioblastoma and metastasis) based on diffusion kurtosis MRI images (DKI) for the assessment of anisotropic changes in brain tissues in the directions from the tumor to the intact (as shown by the standard MRI data) white matter, which will enable generating individual tumor invasion maps.

**Materials and Methods:**

A healthy volunteer and two patients (one with glioblastoma and the other with a single metastasis of small cell lung cancer) were examined by DKI obtaining 12 parametric kurtosis maps for each participant.

**Results:**

During the investigation, we have developed an algorithm of DKI analysis and plotting the profile of tissue parameters in the direction from the tumor towards the unaffected white matter according to the data of standard MRI. Changes of the DKI indicators along the trajectories built using the proposed algorithm in the perifocal zone of glioblastoma and metastasis have been compared in this work. We obtained not only changes in the parameters (gradients in trajectory plots) but also a visual reflection (on color maps) of a known pathomorphology of the process — no significant gradients of DKI parameters were detected in the perifocal metastasis edema, since there was a pure vasogenic edema and no infiltrative component. In glioblastoma, gradients of DKI parameters were found not only in the zone of perifocal edema but beyond the zone of MR signal as well, which is believed to reflect diffusion disorders along the white matter fibers and different degrees of brain tissue infiltration by glioblastoma cells.

**Conclusion:**

The developed algorithm of DKI analysis in brain tumors makes it possible to determine the degree of changes in the tissue microstructure in the perifocal zone of brain glioblastoma relative to the metastasis. The study aimed at obtaining individual maps of tumor invasion, which will be applied in planning neurosurgical and radiation treatment and for predicting directions of further growth of malignant gliomas.

## Introduction

Presently, the direction of research devoted to the study of tumor invasion borders in high-grade malignant gliomas remains topical [1]. Microsurgical removal of the contrast-enhancing tumor part shown by the data of post-contrast T1 images is a current standard of malignant glioma treatment. The perifocal zone of increased MR signal on T2-FLAIR images is an area of the edema infiltrated by tumor cells in glioma. Usually, this zone is not subject to resection and after tumor removal is exposed to radiation therapy [2]. Recently, the term “FLAIRectomy” has appeared meaning tumor removal along the contour of MR signal changes in T2-FLAIR images, i.e. supratotal resection, which is possible only if functionally significant brain zones are preserved [3]. In any case, total surgical removal of glioblastoma seems to be impossible, but the perifocal marginal zone of the postoperative cavity is the primary source of recurrence [1].

At the same time, the investigations including not only MRI but also correlations of MRI and morphological data show that gliomas grade 3–4 spread also beyond the zone of T2 and T2-FLAIR hyperintensity, i.e. the tumor cells may also be in the brain matter shown by standard MRI as unchanged [4–7].

Perifocal edema in metastases, in contrast to gliomas, is considered not to contain tumor cells and is purely vasogenic without the infiltrative component [8].

Different investigations apply various methods and their combinations, radiomics, and artificial intelligence to define tumor borders and differentiation [8–13].

In the previously published work [7], we have analyzed the quantitative diffusion kurtosis and perfusion indicators obtained for the patients with malignant gliomas preoperatively. The study was performed in the zones of contrast-enhancing tumor, perifocal infiltrative edema, and peritumoral intact (as defined by the standard MRI data) brain matter of the affected hemisphere. Additionally, biopsy was taken from these zones, and the obtained indicators were correlated with immunohistochemical and morphological data [7]. The results of this study can be applied for creating individual tumor invasion maps when planning surgical and radiation treatment of patients with brain gliomas and also predicting the direction of further growth of malignant gliomas.

To determine the feasibility of the proposed algorithm for image investigation, we compared the data for patients with glioblastoma and a single metastasis.

**The aim of the study** is to develop and implement an algorithm for image analysis in brain tumors (glioblastoma and metastasis) based on diffusion kurtosis MRI images (DKI) for the assessment of anisotropic changes in brain tissues in the directions from the tumor to the intact white matter as shown by the standard MRI data, which will enable obtaining individual tumor invasion maps.

## Materials and Methods

MRI examinations were performed using the Signa HDxt system (General Electric, USA) with 3.0 T magnetic field strength and an 8-channel head coil generating the following sequences: axial T2 FSE with a 5 mm slice thickness and 1 mm interslice gap; axial T2-FLAIR with a 5 mm slice thickness and 1 mm interslice gap; T1 FSPGR with an isotropic 1×1×1 mm voxel before and after intravenous injection of gadolinium-based contrast agent.

DKI were obtained using echo-planar pulse sequence “spin echo” (SE-EPI) with two diffusion gradients of equal amplitude and duration. The parameters of the obtained images were as follows: 2.5×2.5×2.5 mm voxel; 57 slices; 4621 ms repetition time (TR); 113.3 echo time (ET); 90° Ernst angle; 240 mm field of view (FOV). Diffusion gradients had 60 non-coplanar directions, and the following diffusion factor values: b=0, 1000, and 2500 s/mm^2^. Scanning time was 22 min. It is important to note that DKI is an extension of the classical diffusiontensor MRI and includes an additional image series with b=2500 s/mm^2^.

Patients underwent MRI examination 1–3 days before the operation.

This work presents the MRI studies of one healthy volunteer without pathological changes of the brain and chronic diseases (a 35-year-old man), one patient with glioblastoma (WHO grade 4) in the right frontoinsular area (a 64-year-old man), one patient with the metastasis of small cell lung cancer in the left frontal lobe (a 56-year-old man). The diagnoses were verified by the morphological investigation after microsurgical tumor resection.

The software package for personal computer was written using Python 3.11 (https://www.python.org/) and Matlab v. R2018a (https://www.mathworks.com/) to assess pathological changes in the brain tissues. Diffusion tensor images of the entire head of the patient with brain tumor, the tumor and white matter mask, and the diffusion image-aligned parametric map for the analysis of microstructural changes in the white matter were used as input data. The maps of diffusion and diffusion kurtosis parameters obtained in the process of DKI have been used in the present work: mean diffusivity (MD), fractional anisotropy (FA), mean kurtosis (MK), axial kurtosis (AK), radial kurtosis (RK), kurtosis anisotropy (KA), axonal water fraction (AWF), axial extra-axonal diffusivity (AxEAD), axial intraaxonal diffusivity (AxIAD), radial extra-axonal diffusivity (RadEAD), radial intra-axonal diffusivity (RadIAD), tortuosity of extra-axonal space (TORT).

The study complied with the Declaration of Helsinki (2013) and was approved by the local ethics committee of N.N. Burdenko National Medical Research Center of Neurosurgery (Moscow, Russia). Written informed consent for participation in the study was obtained from all examined people (the healthy volunteer and patients).

## Results

### Algorithm of image analysis

In the process of the investigations, we have developed the algorithm of image analysis and building of tissue parameters in the direction from the tumor to the white matter shown by standard MRI as unchanged, which included several stages.

***I. Segmentation***. At this stage, computation and manual correction of the tumor and white matter mask was conducted. The borders of the tumor and the tumor and perifocal edema in case of the metastasis (or perifocal edema-infiltration in case of glioblastoma) were determined by four image series co-registered between each other: T2, T2-FLAIR, T1-FSPGR before and after contrast enhancement. The term “tumor borders” refers to the enhancing part of the tumor.

Auxiliary programs automatically created tumor and white matter masks, which were further corrected by two radiologists.

It is important to note that segmentation quality influences the results of program operation, since it is very sensitive to the slightest mistakes in delineating the borders of the white matter and tumor.

We failed to find the programs which would perform tumor and white matter segmentation without errors. In this connection, we could not avoid manual correction of the masks in the proposed algorithm of image analysis.

To simplify this process, it was proposed to use auxiliary programs, which automatically generated “rough” masks of the tumor and white matter — correction of the ready images took less time than creation of new ones. Т2, Т2-FLAIR, Т1-FSPGR series were segmented before and after contrast enhancement using CaPTk program version 1.7.6 [14, 15]. The white matter mask was determined by T1 image using Atropos utility from the ANTs program package (https://github.com/ANTsX). Areas of basal nuclei and thalami were removed from the obtained images, since the Atropos program referred these areas to the white matter. The mri_synthseg utility from FreeSurfer was used to define the borders of thalami and basal nuclei [16]. The map of the distance from the tumor border was then computed by the tumor and white matter masks for sections II–IV.

***II. Iterative computation of the “distance map”***. In the first iteration, all voxels adjacent to the tumor are labeled by “1” and only those, which were inside the white matter mask, were left. In the next iteration, voxels adjoining those marked by “1” and inside the white matter mask were labeled by “2”. In the third iteration, voxels adjacent to those marked by “2” and inside the white matter mask and beyond the labeled area of one of the previous iterations or the tumor, were labeled by “3”. Further iterations were performed until the white matter voxels, which could be placed, came to an end.

Let *d_i_*, be the calculated distances of the variable, where *i* is the voxel number. The white matter areas not related to the tumor, which were not marked in any iteration, were excluded from the analysis.

***III. Computation of the matrix of voxel transitions to the tumor border*** considering the apparent diffusion coefficient (ADC), geometry of tumor location, and specificity of the white matter mask.

The following parameters were calculated in each voxel located inside the mask of the white matter:

- ADC along the selected direction *n_ij_* from the voxel *i* to the nearest-neighbor voxel *j*;
- vector of the weight coefficients *W_ij_*, *j*=1…26, by which the direction of transition of voxel *i* to the adjacent voxel *j* was found. Weight coefficients were computed by 26 ADC values in voxels *j*. The weight coefficients are always equal to zero if the distance to the tumor in voxel *j* is greater than in voxel *i*, i.e. *d_j_>d_i_*. In other directions, their value depends on the diffusion properties of the tissue.

The Dipy library for Python (https://dipy.org/) was used in the present work to calculate diffusion tensor and weight coefficients. Data on diffusion tensors in each voxel were converted into the vector with the length 26, whose values are calculated by the formula:

ADCij=Di⋅nij,

where *D_i_* is tensor of diffusion in *i*-s voxel, *n_ij_* are single vectors of directions from voxel *i* to the adjacent voxel *j*.

Only voxels of the white matter were analyzed and for this purpose, white matter and tumor masks were computed and manually corrected. Weight coefficients of trajectory building directions were computed in each voxel of the white matter.

Let us introduce the notations: the voxel with index *i* has 26 adjacent voxels with index *j*, variable *m_ij_* contains weight coefficients for selecting the next voxel for the trajectory. For voxels beyond the white matter mask, the weight coefficients *m_ij_* are equal to zero. Those voxels, in which the value of the distance from the tumor was greater than in the analyzed one, also got zero weight values.

The word “distance” does not denote a geometrical distance calculated along the straight line, but a minimal number of steps (one voxel at a step) inside the white matter mask necessary to reach the tumor margin. In other words, this is the shortest trajectory from a given point to the tumor inside the “labyrinth” of the brain white matter.

***IV. Plotting a pseudorandom trajectory*** for finding the profile of tissue characteristic changes in the directions from the tumor border to the unchanged (according to the standard MRI data) white matter:

- one of the white matter voxels most distant from the tumor on the “map of distances” (at a distance of *d_max* steps) was randomly chosen as a starting point of the trajectory, from which the trajectory was plotted in a stepwise manner in the direction towards the tumor;
- the probability of the transition from voxel *i* to the adjacent voxel *j* at each step was proportional to the weight coefficients *W_ij_*, *j*=1…26;
- when the trajectory reached the tumor margin, the next starting point was chosen at the same distance but where trajectories have not been plotted yet. If there were no such voxels, the next voxel was chosen at a distance of *d_max* steps. The operation was repeated as the starting points were gradually moved closer towards the tumor;
- the operation was repeated until at least 100 trajectories were in each white matter voxel.

The probability of choosing the adjacent voxel with the direction *n_ij_* was calculated by the following formula:

Wij=mij⋅ADCijΣjmij⋅ADCij.

In this way, nvox×26 matrix was calculated, where nvox is a total number of voxels analyzed on the parametric map. The probabilistic coefficients of choosing the next direction while moving from the intact standard MRI-defined white matter to the tumor were counted in this matrix for each voxel. Using these probabilistic coefficients it is impossible to reach “a deadlock” moving iteratively along the white matter despite the geometric complexity of the brain structure.

However, there is a possibility that some trajectories will “wander” at the same distance from the tumor and reach it after too many steps, which will make the process of pseudorandom trajectory computation too long. In order to exclude this situation, there was a parameter in the program limiting that number of successive iterations, when the trajectory may be built not approximating the tumor. In the examples presented in this study, this parameter is equal to 12. We began to compute the trajectories from the starting points representing initially the most distant voxels (at a distance of *n* steps) and through which no trajectories have been built yet. When the trajectory came to the tumor margin, the next starting point was chosen at the same distance, where there were no trajectories. If there were no such voxels, the next voxel was taken at the distance of *d_max-1* steps, through which the trajectories have not yet passed.

The operation repeated as the starting points gradually moved closer to the tumor until voxels without any built trajectories came to an end. Then the cycle repeated from the most remote starting points to the nearest to the tumor. In a new cycle, voxels, in which minimal number of trajectories was built, were chosen for each distance *d_i_*. It should be noted that for the starting point located not at the maximal distance, the trajectory “was stuck” together from two segments: one directed towards the tumor border and the other coming out from the starting point in the direction opposite to the tumor. To build the second segment, the matrix of the probabilistic coefficients of choosing directions with the size of nvox×26 was computed. This matrix was computed similarly to the matrix used for the first segment but in the matrix for the second segment, on the contrary, all adjacent voxels, in which the distance to the tumor was smaller than the distance to it from the analyzed voxel, were nullified.

The cycle of plotting pseudorandom trajectories to the tumor was considered completed when there were minimum of 100 generated trajectories in each white matter voxel. It takes about 5 min for the modern 16- core CPU computers to perform the cycles in Matlab environment in a multiflow mode.

***V. Plotting tissue characteristic profiles along the trajectories:***

**Figure 1.**
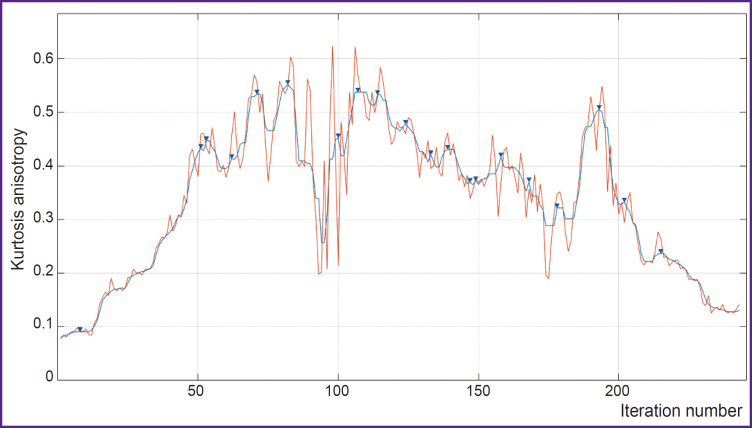
Profile of kurtosis anisotropy parameter changes along the trajectory built from the glioblastoma border (at the beginning of coordinates) towards the unchanged (by the standard MRI data) white matter Red color shows the plot built according to the initial data, blue is the profile smoothed by the 6^th^ grade median filter. Arrows show local maximums

- profiles were built along the generated trajectories, i.e. plots of numeric changes in voxels on the selected parametric DKI map depending on the distance to the tumor margin (Figure 1);
- a 6^th^ degree median filter was used to smooth small “noise” peaks in the profiles (see Figure 1);
- extremums and areas of gradients from minimums to maximums or from maximums to minimums were marked on the smoothed profile.

Each trajectory was separately analyzed. The algorithm, initially proposed in this work, was tested on the parametric kurtosis maps, which are sensitive to the effect of thermal noise artifacts. An example of the profile obtained by the map of kurtosis anisotropy is presented in Figure 1. A large number of local peaks, which appeared probably due to the thermal noise, are seen. These noise peaks may be removed by a median filter. Empirically, an optimal order of this filter equal to 6 was selected. One of the advantages of image analysis using pseudorandom trajectories is the possibility of smoothing the data of the analyzed parametric map and complete exclusion of the effect of the neighboring basal nuclei and cortex (grey matter) and the cerebrospinal fluidcontaining structures (ventricles and subarachnoid spaces) on the result of MR signal.

***VI. Plotting the parametrical map of “gradients” for the analyzed tissue parameter.*** The following statistics were computed using the analyzed profiles for each voxel of the white matter: *P* trajectories in the given voxel had an area of significant gradient, a total of *Q* trajectories have been built in the given voxel. The parametric map was calculated by the 100%(*P*/*Q*) formula, which “highlighted” the significantly changed areas of the analyzed DKI parameter in the direction from the tumor to the intact white matter as shown by the standard MRI data.

After smoothing, extremums and rising and falling segments of the signal from the voxels, across which the trajectory passed, were marked on each profile. After this operation for all obtained trajectories, each of the analyzed voxel was assigned three values: 1) a number of trajectories in the given voxel on the profile, which contained an area of increased signal; 2) total number of trajectories in the given voxel with the area of decreased signal on the profile; 3) total number of trajectories built through the given voxel. Having divided the values from the first or second point by the total number of trajectories in the voxel, images may be obtained, where there are marked areas of significant value gradient for the used parameter near the tumor, which may be overlooked on the parametric map during visual analysis.

### Clinical testing of the algorithm

**Figure 2.**
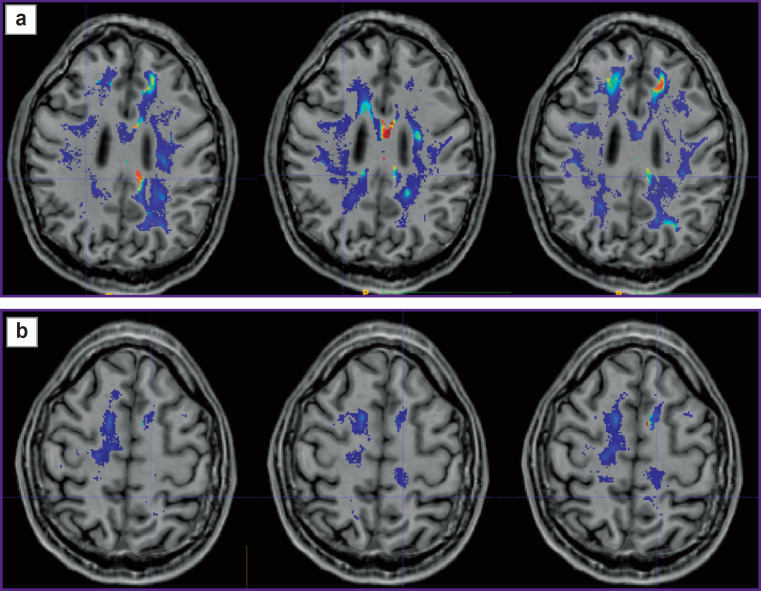
Alignment of the parametric maps of diffusion kurtosis images, obtained from the healthy volunteer (from left to right: MK, KA, AWF) with T1- FSPGR images Color highlights the areas of value gradients in the healthy volunteer after the application of glioblastoma (а) and metastasis (b) masks

**Figure 3.**
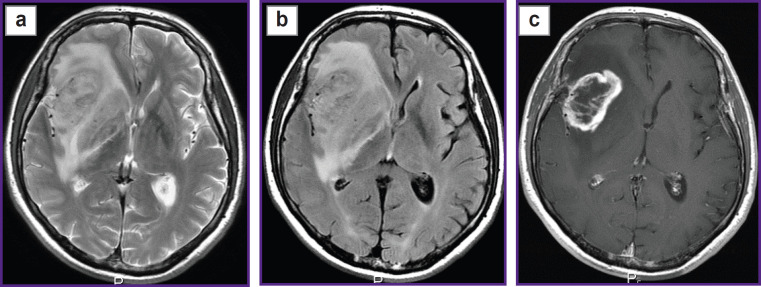
Patient with glioblastoma in the right fronto-insular region: (а) Т2-weighted MRI, (b) Т2-FLAIR images, (c) contrast-enhanced Т1

**Figure 4.**
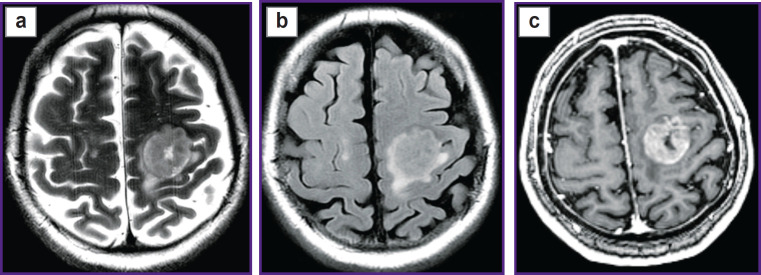
Patient with metastasis of small cell lung cancer in the left posterio-frontal region: (а) Т2-weighted MRI, (b) T2-FLAIR images, (c) contrast-enhanced Т1

Prospective application of the algorithm was tested on one healthy volunteer (Figure 2) and two patients: with glioblastoma, WHO grade 4 (Figure 3), and with a single metastasis of small cell cancer (Figure 4).

The tumor localization in the healthy volunteer was simulated. The patient’s tumor mask was transferred on the white matter mask of the healthy volunteer using transformation files. These files were obtained after the alignment of T1 images of each patient and T1 image of the volunteer. The volunteer’s parametric maps were not modified, changes were made only to the file with the white matter: the tumor structures were added in this mask such as necrosis, contrast-enhancing part, and perifocal edema (or infiltrative edema for glioblastoma). Summarizing the above paragraph IV, we may conclude that the location of pseudorandom trajectories depends on the geometry of the white matter, the shape and localization of the tumor, and diffusion tensors in the white matter of the examined individual. Thus, these trajectories in the patient and healthy volunteer with the simulated tumor should be similar.

**Figure 5.**
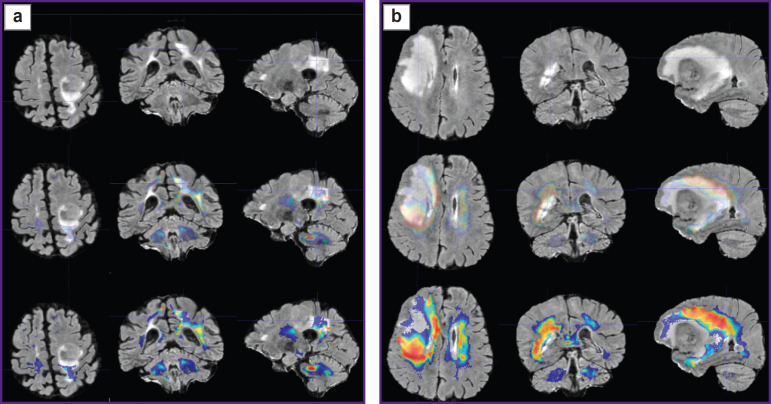
Plotting the maps of value gradients of mean kurtosis (МК) in patient with glioblastoma (а) and in patient with metastasis (b) The upper row — T2-FLAIR images in three projections; middle row — comparison of T2-FLAIR with MK parametric map (semitransparent for better contour visualization of the changed MR signal by T2-FLAIR); lower row — comparison of T2- FLAIR with MK parametric map (in full color, where red and yellow tones show the zone of greatest parameter values)

**Figure 6.**
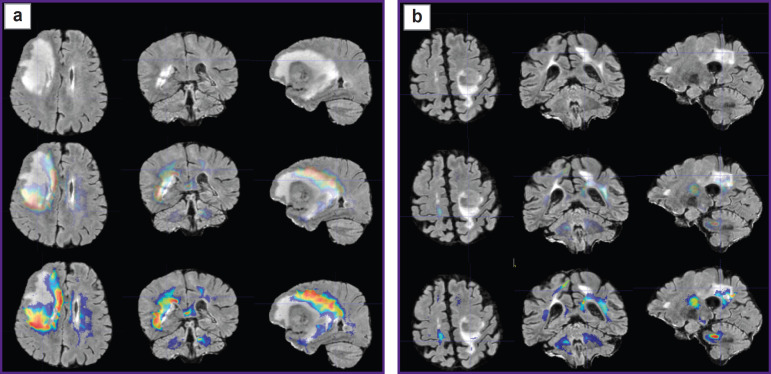
Plotting the maps of value gradients of the axonal water fraction (AWF) in patient with glioblastoma (а) and patient with metastasis (b) Upper row —T2-FLAIR images in three projections; middle row — comparison of T2-FLAIR with AWF parametric map (semitransparent for better contour visualization of the changed MR signal by T2-FLAIR); lower row — comparison of T2- FLAIR with AWF parametric map (in full color, where red and yellow tones show the zone of greatest parameter values)

**Figure 7.**
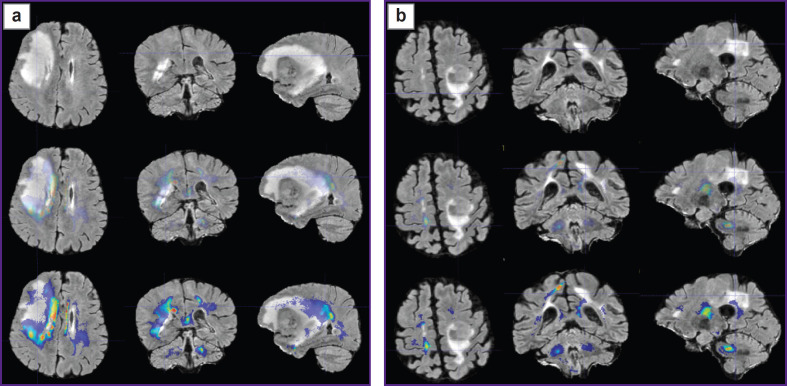
Plotting the maps of value gradients of kurtosis anisotropy (KA) in patient with glioblastoma (а) and patient with metastasis (b) Upper row — T2-FLAIR images in three projections; middle row — comparison of T2-FLAIR with KA parametric map (semitransparent for better contour visualization of the changed MR signal by T2-FLAIR); lower row — comparison of T2- FLAIR with KA parametric map (in full color, where red and yellow tones show the zone of greatest parameter values)

To visualize the changes in the white matter, DKI were successively analyzed by all parameters indicated in the section “Materials and Methods”. The most important changes appeared to be on the MK, RK, AWF, KA, AK maps. Moreover, parameter gradients were not found beyond the part of the enhancing tumor in the patient with the metastasis, whereas in the patient with glioblastoma, the zone of parameter changes extended to the perifocal infiltrative edema and even further. Examples for the MK, AWF, КА parameters are given in Figures 5–7.

## Discussion

In our previous work, we have shown high sensitivity and specificity of diffusion kurtosis MRI parameters when differentiated the infiltrative edema zone in malignant gliomas from perifocal white matter seen as intact on standard MRI, which was confirmed by the data of biopsy and correlation with Ki-67 and Bcl2 markers [7]. Continuing this study, we assumed that the obtained data can be applied for plotting the maps showing changes in the DKI parameters not only in the zones of interest but in all obtained brain slices and also along the periphery of the pathological site, which will probably reflect the degree of tissue infiltration by the tumor cells.

The advantage of the proposed method of image analysis consists in objectivization of insignificant increase in the brightness of voxels of the parametric DKI maps in determining the spread of tumor invasion in the perifocal zone of glioblastoma in comparison with the metastasis.

**Figure 8.**
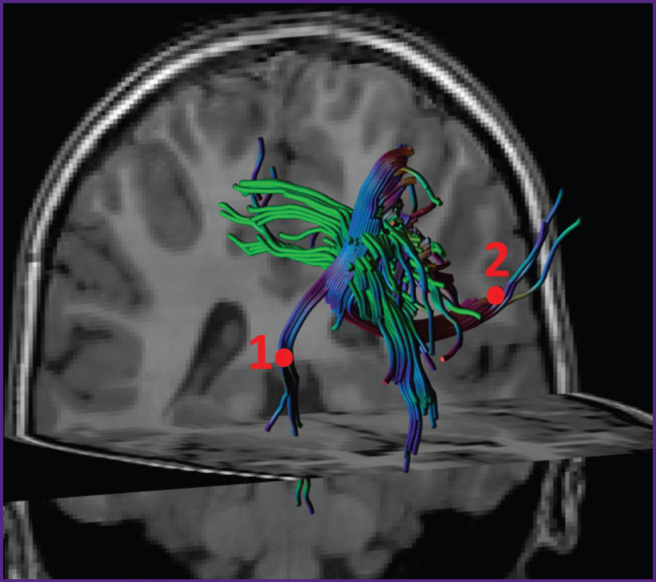
Example of reconstructed crossing tracts, along which tumor cells may spread from zone 1 to zone 2 The trajectory along the tracts from zone 1 to zone 2 does not fit into the plane of any single slice, therefore, changes in the tissue signal along this trajectory and localization of the boundary of tumor cell spreading are impossible to define by radiologic images

It is known that the main pathway of glioblastoma cell spreading in the brain is along myelinated axons [1, 17–19]. We have supposed that voxel brightness in the white matter on the parametric DKI maps is proportional to the concentration of the tumor cells in this voxel. Let us consider two areas of interest located on one slice of the parametric map. The first area is the zone of infiltrative edema in the white matter, where the concentration of tumor cells may be high [1]. The second area is located in the white matter significantly far from the tumor, where presumably the tumor cells are absent. If we assume that the tumor cells from the first zone of interest were spreading within one slice, then it is possible to see the tumor border by the gradient picture having selected such visualization settings that the contrast is visually distinguishable. If the same two zones of interest are located on different slices and the tumor cells were spreading along the complex trajectory of the intersecting or osculating fibers, as in Figure 8, in this case it is impossible to define visually the border of the growing tumor. We investigated the gradients of the DKI parameters along the trajectories in all directions.

In our work, we have compared how the indicator changed along the trajectories generated by means of the proposed algorithm in the perifocal zone of glioblastoma and metastasis. We obtained not only changes of the parameters (gradients in the trajectory plots) but also a visual reflection (on color maps) of the known process pathomorphology: no significant gradients of the DKI parameters were found in the perifocal metastasis edema, since there was a pure vasogenic edema and no infiltrative component. In glioblastoma, gradients of DKI parameters were detected not only in the zone of perifocal edema but also beyond the limits of the zone of standard MR signal changes, which is believed by us to reflect the diffusion disorder along the white matter fibers and various degree of brain tissue infiltration by the glioblastoma cells.

Different authors [20–22] have studied perifocal tumor edema using DKI in glioblastoma and metastases. These investigations have shown reliable differences of kurtosis parameters and the possibility to differentiate pure from infiltrative edema. This differentiation is necessary for establishing the correct diagnosis in case of a single large metastases simulating glioblastomas (or vice versa). These investigations are also interesting from the point of view of fundamental science. In our work, we visualized the differences between the perifocal zone of glioblastoma and metastasis on parametric color maps generated with our algorithm.

If the structure and pathophysiology of glioblastomas have somehow been studied, the perifocal zone, including edema and peritumoral intact brain matter, is weakly explored [1, 18, 19]. Since this topic is very important, investigations using various methods and their combinations are being carried out. For example, the perifocal zone of glioblastomas was studied [9] to predict a further tumor growth using the multiparametric analysis in different MRI modes including also diffusion tensor MRI. This work has shown a heterogeneous structure of the peritumoral area and the signs of tumor infiltration, which may reflect subsequent direction of tumor spreading, which, however, has not been proved by follow-up MRI.

Diffusion kurtosis MRI gives non-Gaussian parameters of water diffusion, which probably reflect a complex tissue microstructure most correctly relative to other quantitative methods of neuroimaging [22–25]. In this connection, we used the DKI parameters considering them to be most sensitive for fine changes in the structure of brain tissue.

Delgado et al. [26], investigating patients with gliomas grade 2 and 3 using diffusion kurtosis MRI, have found a significant difference of parameters in the peritumoral white matter and in the white matter of the contralateral hemisphere (without tumor growth signs), which is in line with the data of our previous study performed for high grade gliomas [7].

In the perifocal zone of the contrasted part of glioma, the DKI parameters are changing due to the presence of vasogenic edema with tumor infiltration, while beyond the pathologically increased MR signal, according to T2- FLAIR, alteration of the DKI parameters, very sensitive to structural changes, is probably due to the infiltration of the tissue by the tumor cells [7]. This is reflected on the profiles of kurtosis parameters along the trajectories generated by means of our algorithm.

There is a small number of works comparing the results of MRI examinations, and histological and immunohistochemical data beyond the visible glioma boundaries. These investigations have shown that tumor cells may be detected at a significant distance from the main tumor mass, even in the normal brain matter as defined by a standard MRI [5, 6, 27–29].

Diffusion-tensor images of the entire head of the patient with brain tumor as well as the parametric DKI maps aligned with the diffusion image, employed for analyzing the microstructural changes in the white matter, were used as input data for the program presented in this work. Images of tissue T1- or T2-relaxation times (in MR relaxometry), PET images, perfusion maps, and so on, may also be such parametric maps.

The algorithm suggested in this work will be used soon on a large group of patients with gliomas grade 2–4.

**The study limitation** lies in the impossibility of verifying morphological changes over the entire tumor contour, verification is possible only in a restricted number of zones of interest. For example, earlier we have investigated the biopsy materials only from three zones of interest [7].

DKI are very sensible to various kinds of artifacts, which places increased demands on the quality of equipment and data post-processing.

The manual correction of images also may be referred to the drawback of the study.

The data obtained require validation in the form of MRI examinations in dynamics for analyzing further direction of recurrent tumor growth.

## Conclusion

The algorithm developed on the basis of diffusion kurtosis MRI for image analysis in brain tumors makes it possible to assess anisotropic changes in the brain tissue in the direction from the tumor towards the intact white matter as defined by the standard MRI data and thus to determine the degree of microstructure changes in the perifocal zone of brain glioblastoma relative to a metastasis. The study was carried out to obtain individual maps of tumor invasion, which will be useful for planning neurosurgical and radiation therapy and also for predicting the directions of further malignant growth of gliomas.
